# Where We Mentalize: Main Cortical Areas Involved in Mentalization

**DOI:** 10.3389/fneur.2021.712532

**Published:** 2021-08-27

**Authors:** Matteo Monticelli, Pietro Zeppa, Marco Mammi, Federica Penner, Antonio Melcarne, Francesco Zenga, Diego Garbossa

**Affiliations:** Neurosurgery Unit, Department of Neuroscience “Rita Levi Montalcini,” Turin University, Turin, Italy

**Keywords:** mentalization, human brain, cortical areas, functional anatomy, practical neuroscience

## Abstract

When discussing “mentalization,” we refer to a very special ability that only humans and few species of great apes possess: the ability to think about themselves and to represent in their mind their own mental state, attitudes, and beliefs and those of others. In this review, a summary of the main cortical areas involved in mentalization is presented. A thorough literature search using PubMed MEDLINE database was performed. The search terms “cognition,” “metacognition,” “mentalization,” “direct electrical stimulation,” “theory of mind,” and their synonyms were combined with “prefrontal cortex,” “temporo-parietal junction,” “parietal cortex,” “inferior frontal gyrus,” “cingulate gyrus,” and the names of other cortical areas to extract relevant published papers. Non-English publications were excluded. Data were extracted and analyzed in a qualitative manner. It is the authors' belief that knowledge of the neural substrate of metacognition is essential not only for the “neuroscientist” but also for the “practical neuroscientist” (i.e., the neurosurgeon), in order to better understand the pathophysiology of mentalizing dysfunctions in brain pathologies, especially those in which integrity of cortical areas or white matter connectivity is compromised. Furthermore, in the context of neuro-oncological surgery, understanding the anatomical structures involved in the theory of mind can help the neurosurgeon obtain a wider and safer resection. Though beyond of the scope of this paper, an important but unresolved issue concerns the long-range white matter connections that unify these cortical areas and that may be themselves involved in neural information processing.

## Introduction

When discussing “mentalization,” we refer to a very special ability that only humans and, to the present knowledge, few species of great apes have. It is the ability of thinking about themselves and to represent in their mind their own mental state, attitudes, and beliefs and also those of others (1). Some authors have called this peculiar skill “theory of mind,” defined as an awareness of the likely content of other people's minds (2).

It is now well-known that mentalizing is not a unitary process but that it assumes a wide variety of known and unknown, and specific and unspecific subprocesses such as emotions, inferential reasoning, understanding of causality, and the distinction between self and other.

A large number of neuroimaging and lesion studies have attempted to clarify the neural substrate underlying mentalization. Moreover, observations made by numerous authors during intraoperative mapping have been added in the last decades. Awake surgery, with the possibility of direct electrical stimulation (DES) of the brain, provided neurosurgeons a crucial opportunity to better comprehend and study these networks and areas *in vivo*. Intraoperative DES temporarily alters the function of the stimulated area and thus provides real-time anatomo-functional correlations with high spatial resolution. It has been demonstrated that data acquired through this technique are highly specific and mostly match results gained with other neuroscientific approaches (3). To date, DES is the only technique allowing direct gain of causal information on the functional role of cortical areas as well as white matter tracts in cognition and behavior (4).

**Figure 1:**
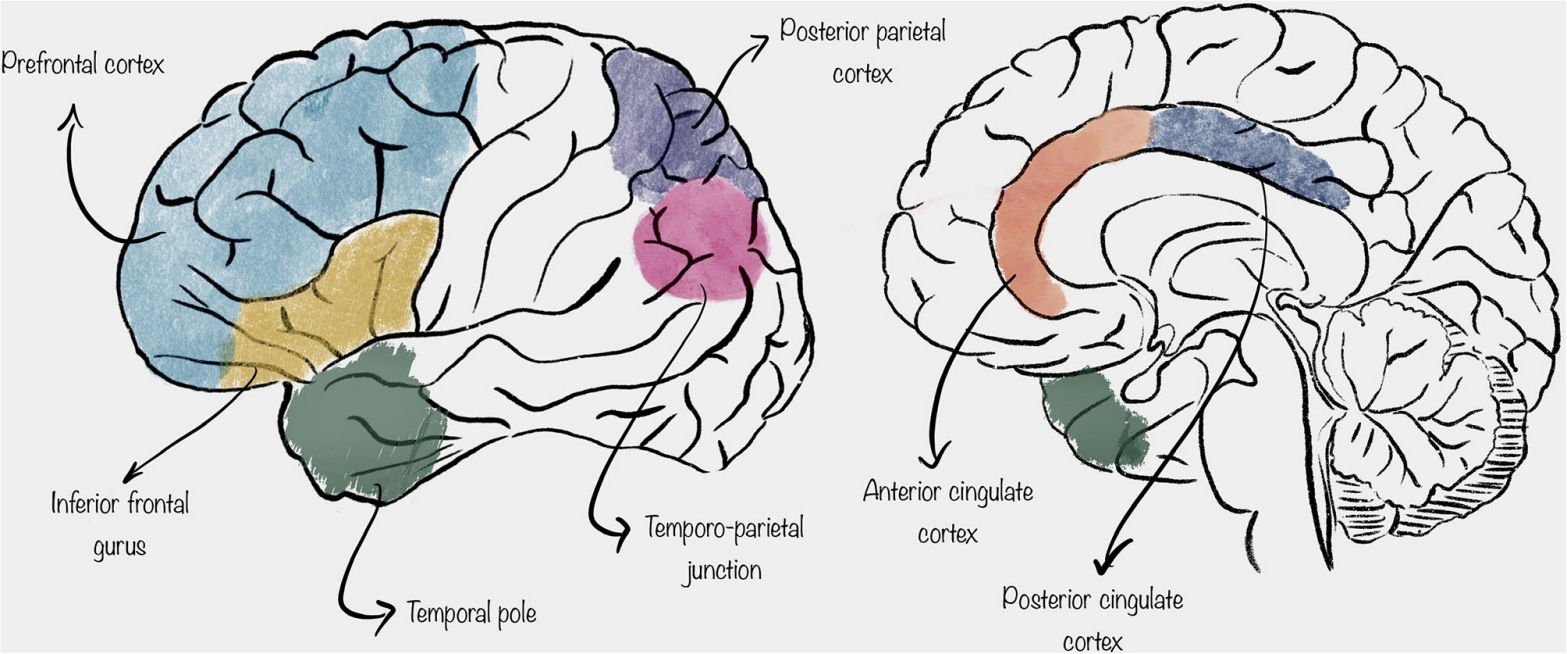
Schematic representation of the location of the cortical areas described in this review.

Thanks to the observation made with the aforementioned methods, it has been suggested that mentalization is carried out by an extensive network of spatially distributed cortical areas, mainly including the prefrontal cortex (PFC), the inferior frontal gyrus (IFG), the temporo-parietal junction (TPJ), the posterior parietal cortex (PPC), the temporal pole, and the cingulate cortex (4, 5) (Figure 1).

In this review, a summary of the main cortical areas involved in the mentalization process is presented. It is the authors' belief that knowledge of the neural substrate of metacognition is essential not only for “pure neuroscientists” but also for “practical neuroscientists” (i.e., neurosurgeons), in order to better understand the pathophysiology of mentalizing dysfunctions in brain pathologies, especially those in which integrity of cortical areas is compromised. Furthermore, in the context of neuro-oncological surgery, understanding the anatomical areas involved in mentalization can help neurosurgeons to obtain a wider and safer resection, avoiding surgical injury to those structures.

Though beyond of the scope of this paper, an important but unresolved issue concerns the long-range white matter connections unifying these cortical areas, that may be themselves involved not only in carrying information but also in processing neural information (6, 7).

## Materials and Methods

A thorough literature search using PubMed MEDLINE database was performed. The search terms “cognition,” “metacognition,” “mentalization,” “direct electrical stimulation,” “theory of mind,” and their synonyms were combined with “prefrontal cortex,” “temporo-parietal junction,” “parietal cortex,” “inferior frontal gyrus,” “cingulate cortex,” and the names of other cortical areas to extract relevant published papers. Initial screening was performed by reading the abstract; only the papers that qualified from this step were analyzed in their entirety. References of extracted papers were screened for other relevant studies to include. All study designs were included in this non-systematic review. Non-English publications were excluded. Data were extracted and analyzed in a qualitative manner.

## Prefrontal Cortex

It is well-known, from years of literature about this topic, that the PFC is one of the most involved cortical areas in mentalization. Recent research in cognitive neuroscience has clearly demonstrated how it is implicated in higher cognitive mechanisms (8, 9). These findings are consistent with the hypothesis that the PFC is a key structure, with a crucial role in generating the relevant kind of higher-order cognitive states that underlie phenomenal awareness.

PFC is a generic concept that encloses subregions that are anatomically and functionally different. In 2005 in his *Principles of Brain Evolution*, Striedter proposed that the PFC could be divided into two different regions from a functional and anatomic point of view and according to the evolutional development (9). The first region is the ventromedial PFC, which is composed of the ventral PFC and the medial PFC; these cortices are present in all mammals. The second is the lateral PFC, which is composed of the dorsolateral PFC and the ventrolateral PFC, present only in primates. Also at a microscopic level, the PFC has several different subregions with different cytoarchitectonic properties (granular, dysgranular, or agranular); different patterns of connectivity between each other and with sensory, memory, and conceptual processing regions; and different phylogenetic histories (10, 11).

Regarding neurophysiological activity, PFC neurons do not directly discharge an electric impulse responding to constant stimuli; instead, neuronal coding could be complex and involve local and distributed ensembles within and between areas of the PFC (12).

Lau and Rosenthal in their 2011 paper proposed an interesting theory about the higher-order representations in the PFC (13). Their work was primarily about visual perception; however, expanding the discussion to the ventral lateral PFC, they generalized their model to other external senses (14). In this model, different roles for specific areas of the PFC were identified. Specifically, the dorsolateral and the polar regions of the PFC were identified as key areas. The authors suggested that higher-order representations in the prefrontal network are not themselves consciously experienced but facilitate the experience of first-order states. The latter are defined as those states depending on sensory input that allow us to respond meaningfully to the perception of an external object. In their model, conscious experience of the higher-order representation requires an additional level of higher-order representation, whose location was not identified by Lau and Rosenthal.

Other authors suggested that the polar prefrontal region might be responsible for additional higher-order representations (15). For example, medial and insular prefrontal areas receive inputs from conceptual, memory, and subcortical circuits, as well as from sensory and perceptual networks, and, in turn, connect with lateral–polar prefrontal areas. In other words, these prefrontal areas may construct lower-order representations used by the lateral–polar prefrontal areas in the assembly of higher-order representations that are phenomenally experienced. Another possibility is that the higher-order network does not involve a fixed set of prefrontal areas but instead a coalition of areas and connections that are flexibly recruited on a situational basis to meet the needs of the moment.

## Inferior Frontal Gyrus

The IFG is the lowest part of the lateral frontal lobe. From a topographic point of view, it can be divided into three distinct region called the pars opercularis, pars triangularis, and pars orbitalis (16). These three portions are macroscopically divided by two sulci arising from the Sylvian fissure: the ascending ramus separates the pars opercularis and pars triangularis, while the horizontal ramus lies between the pars triangularis and pars orbitalis.

Many functional magnetic resonance imaging (fMRI) studies, as well as DES studies performed during awake craniotomies, indicate that the IFG might be a paramount mentalization site (17, 18). In particular, the lateral part of the pars opercularis has been identified as a key structure in mentalizing accuracy, and some authors proposed that the impairment in mentalizing elicited by DES during awake surgery could be related to the transient inactivation of a mirror system. Mirror systems are, to the present knowledge, the main neuronal substrate underlying the human ability to interact with others and to understand the actions of others without logical inferences (18).

Indeed, the presence of neurons with mirror properties in the pars opercularis is well-established. Most fMRI studies that deal with tasks targeting the mirror system have pointed out this brain region as a central cortical node (19, 20). It is also known that damage to the pars opercularis impairs mirror-related functions, such as emotional empathy and face-based perceptual mentalizing (21). Moreover, abnormal neural activity within the mirror network (and especially within the pars opercularis) is one of the neural bases that characterize some psychopathological conditions (such as autistic spectrum disorders), with huge impairment in social perception and cognition, including mentalizing (22).

As suggested by many papers, while a mirror dysfunction-based mechanism arguably depends on the function of the pars opercularis, the same interpretation becomes less obvious for the adjacent pars triangularis, which is not generally considered to be a mirror area, although several fMRI studies indicate the opposite (19, 20, 23). The reason why the pars triangularis is not considered a mirror area is that its neurons, at least in its dorsal portion, discharge during action and emotional face perception (19), but they do not seem to be activated during execution, a necessary condition for classification as mirror neurons by their original definition provided by Rizzolatti and colleagues (24). To the authors' best knowledge, only one study has currently provided evidence to suggest that imitation and observation of faces expressing emotional states activate the pars triangularis, as well as the classical anterior mirror areas (e.g., the pars opercularis and the ventral premotor cortex) (25).

## Temporo-Parietal Junction

A large body of evidence, mainly from functional neuroimaging studies, highlights that the entire parietal cortex is not equally involved in mentalization processes, which depends on the coordinated interaction between the medial PFC and the posterior temporal gyrus at the junction with the parietal cortex. Because of its anatomical location, this region was termed the TPJ.

Many papers published in the literature, both fMRI studies and “lesion” studies performed during neurosurgical operations using DES have shown that the inferior part of the parietal cortex, more frequently on the right side, at the junction with the posterior temporal cortex, plays a critical role in matching signals arising from self-produced actions with signals from the environment (26, 27).

Intraoperative studies using DES during awake craniotomies once again provided similar results to neuroimaging studies, detecting in the TPJ a key mentalizing structure (28). Such function has usually been located with the “reading the mind in the eyes test” (RME) during awake surgery.

## Posterior Parietal Cortex

The PPC is a set of interconnected areas located just rostrally to the TPJ, and it is one of the major associative regions of the mammalian brain, receiving multisensory inputs (visual, auditory, somatosensory, and vestibular) (29). Integration of these multiple perception signals allows a higher-level representation of self and of peripersonal space. Moreover, multisensory input association is the basis of movement-related decision making, which requires rapid integration of sensory stimuli with presumed consequences of actions, based on memory of previous similar situations (30).

Many authors suggest that in non-human primates, the PPC can be divided into distinct subregions. The first is the lateral intraparietal area, which contains high concentrations of neurons responsive to saccades and is involved in perceptual decision making. Others subregions are the parietal reach region, involved in reaching actions, and the anterior intraparietal area, linked to grasping movements (31, 32). Each PPC subregion significantly modulates the encoding and processing of the other subregions (33).

Since making right decisions requires choosing relevant information from competing distractors, it is likely that the PPC benefits from the experience of past decisions. In this regard, there are studies that highlight how lateral intraparietal neurons code the consequences on sensory perceptions produced by a saccade (34, 35). In non-human primates, similar representations for hand-reaching actions were found (36). In humans, a transcranial magnetic stimulation (TMS) study demonstrated that functional inactivation of the right and left PPCs resulted in biased saccades (35).

Taken together, these studies suggest that a fundamental role of the PPC is to filter sensory inputs, focusing attention on behaviorally relevant stimuli in a top-down fashion. The contextual relevance of a given stimulus is determined on the basis of past experience. PPC neural networks therefore constitute a memory substrate for perceptual decision making (37). As in many others cortical networks, PPC neurons show selective tuning, such as a selective response to the direction of saccades in the lateral intraparietal area. It can therefore be assumed that the repeated activation of certain successful response networks may facilitate their subsequent activation and thus constitute the substrate of the experiential component of decision making.

Most research regarding PPC decision making has been related to perceptual decisions. However, it is presumable that this cortical region is also involved in the integration of stimuli and evaluations of non-motor origin, such as emotions and the social context (38). The role of the PPC in the perceptual decision-making processes implies its involvement in the mentalization process, although its role seems to be less relevant than that of other regions considered in this review (39).

## Temporal Pole

As for the structures described above, available evidence suggests that damage occurring at the level of temporal poles can impair the ability to mentalize, as described by Funnell (40). Funnell's findings are in agreement with those from Damasio's group that suggested that the temporal poles are convergence zones, where simpler features from different modalities are put together to define, by their conjunction, unique individuals and situation knowledge (41). It seems to be exactly this convergence of information that gives us the ability to understand the function of an object that we are looking at and the chance to infer not only its potential at the moment but also how that object could be modified by the context in which it appears (42). It is therefore clear that these processes generated at the temporal poles have their relevance in the mentalizing mechanism, especially in order to provide the so-called moment-to-moment knowledge about a specific object in a specific condition (43).

Also in this case, DES studies exist with results comparable with the ones obtained by neuroimaging (44).

## Cingulate Cortex

### Anterior Cingulate Cortex

The anterior cingulate cortex (ACC) is located near the medial aspect of the frontal lobe and can be differentiated from the posterior cingulate cortex (PCC) on the basis of cytoarchitecture patterns of projections, as well as function. The anterior portion of cingulate cortex appears to be tasked with executive functions, whereas the posterior part of the cingulum by evaluative functions (43). Several studies based on animal models have shown that ACC lesions resulted in decreased levels of social interaction and decreased preference for social stimuli (45–48). Human fMRI and DES studies have confirmed that ACC activity increased when patients were asked to evaluate others' decisions in a social context and in mentalizing processes in general (49–52).

In addition, the ACC is known for its role, not only in social information coding but also in the processing of information that guides decisions in daily routine (53, 54). Studies based on single-unit recording patterns carried out in non-human primates demonstrated that neuron populations in the ACC were enrolled by a wide range of stimuli, mostly reward related, that led to optimize the decision-making process (55–57).

Matsumoto et al. described that, in addition to all of that above-reported functions, some ACC cell populations showed responses according to errors in the prediction of positive or negative outcomes of some proposed scenarios (58). This was proven to be useful to subjects during the execution of trial-and-error tasks, in order to reach an optimal decision.

Another proposed function that involves the ACC concerns the understanding of others' decision making through simulation. It has been proposed that the neural circuitry used for one's own decision-making process is also used for the understanding of the decisions of others (59), in a process called embodied simulation, which has been confirmed for the understanding the actions of others (60–63). If understanding others' decisions occurs through simulation, it is possible that the same areas of the ACC may be used to process errors in one's own or another's prediction upon receiving informative feedback.

### Posterior Cingulate Cortex

The PCC is located near the medial aspect of the inferior parietal lobe (64). According to Vogt's model, the PCC is composed of Brodmann areas 23 and 31; it is delimited superiorly by the marginal ramus of the cingulate sulcus, inferiorly by the corpus callosum (CC), posteriorly by the parieto-occipital sulcus, and anteriorly by Brodmann area 24, the midcingulate region (65). From a cytoarchitectonic point of view, the PCC contains paralimbic cortex, with transitional features between the typical six layered isocortex and the primitive allocortex of true limbic structures (66).

Much of the information that we have today about structural connectivity and functions of the PCC is assumed from non-human primate studies. However, during the last decades, the continuous improvement of MRI/fMRI techniques has led to a better *in vivo* comprehension of the PCC in humans (67, 68). Diffusor tensor imaging (DTI) tractography, for instance, has confirmed the presence of connections between the ventral part of PCC and the retrosplenial cortex and the medial temporal lobes, as well as connections from the dorsal part of PCC to the ventromedial PFC along the cingulum (69). Some speculations have been made regarding the functions of these connections, but a thorough comprehension of these networks in humans is still elusive. However, it seems that these connections play a key role in information processing and integration (68).

## Discussion

Since the beginning of history, human beings have been obsessed with questions about their nature, about their relationship with the world, and of course about consciousness. Consciousness is, as defined by the Oxford Living Dictionary, “the state of being aware of and responsive to one's surroundings,” and it is a state that unites in this awareness both humans and many animal species, who are definitely well-aware and responsive to their surrounding environment. However, humans (and very few great apes, to present knowledge) have another very special feature that diversifies them from the majority of other animals: the ability to mentalize, defined as the skill of thinking about themselves and to represent in their mind, not only their own mental state but also the mental state of others. Nowadays, it is well-understood that mentalizing is not a unitary task, but it assumes a wide variety of diverse subprocesses.

It is not trivial to highlight that, when discussing “brain functions,” we must not restrict them only to motor and language functions, which are undoubtedly crucial to maintain a good quality of life but are not sufficient. The human being is also made of self-awareness, beliefs, and representation of one's own and others' mental state: in one word, mentalization.

In this brief review, we reported the main cortical areas involved in this fascinating but still widely mysterious process, in order to provide a quite simple but precise idea of their spatial representation for neurosurgical usage.

Knowing that the prefrontal areas, IFG, TPJ, or temporal pole (whatever the side and whatever the so-called “hemispheric dominance”) are involved in higher-level processes must lead brain surgeons to be cautious even if, or perhaps mostly when, the so-called “right non-dominant hemisphere” is involved, in order to avoid permanent deficits of the patient's higher-level functions. And that is possible only knowing where and also how to intraoperatively test those functions. The most accurate way to achieve this goal is to create a real-time cortical brain map using DES and appropriate tasks given to the patient who has to be awake before corticectomy is performed. Indeed, nowadays, it is mandatory for brain surgeons to shift the paradigm from “anatomical based resection” to “functional based resection,” being aware of functional limits and areas, mastering awake surgery and DES techniques, at least when approaching low-grade tumors or other pathologies with a long survival rate (e.g., cavernomas) and in which, precisely for this reason, postoperative quality of life is crucial.

It is not the purpose of this article to show and explain the indications and technique for awake surgery craniotomy or DES, but we need to underline, once again, how these have contributed to improve our still limited knowledge about brain function. The subject is still largely unknown, and more studies are needed in order to extend our understanding of this remarkable function.

Finally, it must be underlined again that another important but unresolved issue concerning the mentalization process regards the long-range white matter connections, which unify mentioned cortical areas. Such white fibers may be themselves involved in neural information processing; it is an issue of paramount importance, and more studies are needed in a “connectomic” view of a functional and plastic brain.

The main limit of this paper is its non-systematic nature; we tried to be as concise as possible in order to give young neurosurgeons or neurosurgeons who want to approach brain surgery a schematic view and a basic knowledge of which cortical areas are involved in mentalization. Another limitation is that we have, consciously, left out all the world of subcortical white fiber tracts, focusing our work only on gray matter cortical areas.

## Conclusions

The mentalizing process is still a wide and poorly understood field in cognitive neuroscience. A few publications exist concerning this topic applied to neurosurgery. In this brief review, we reported the main cortical areas involved in this peculiar human skill, namely, the PFC, TPJ, PPC, temporal poles, and cingulate cortex.

We strongly believe that brain surgeons cannot ignore this topic especially when the patient's postoperative life expectancy is long and a return to normal life is anticipated: when damaged, these areas could lead to permanent deficits of the patient's higher-level functions, precluding many normal life nuances.

## Author Contributions

MMo: idea and text writing. PZ and MMa: data collection. FP: text writing. AM: supervision. FZ and DG: manuscript correction. All authors contributed to the article and approved the submitted version.

## Conflict of Interest

The authors declare that the research was conducted in the absence of any commercial or financial relationships that could be construed as a potential conflict of interest.

## Publisher's Note

All claims expressed in this article are solely those of the authors and do not necessarily represent those of their affiliated organizations, or those of the publisher, the editors and the reviewers. Any product that may be evaluated in this article, or claim that may be made by its manufacturer, is not guaranteed or endorsed by the publisher.
